# Predicting Long-Term Engagement in mHealth Apps: Comparative Study of Engagement Indices

**DOI:** 10.2196/59444

**Published:** 2024-09-09

**Authors:** Yae Won Tak, Jong Won Lee, Junetae Kim, Yura Lee

**Affiliations:** 1 Department of Information Medicine, Asan Medical Center University of Ulsan College of Medicine Seoul Republic of Korea; 2 Division of Breast Surgery, Department of Surgery, Asan Medical Center University of Ulsan College of Medicine Seoul Republic of Korea; 3 Graduate School of Cancer Science and Policy National Cancer Center Goyang-si Republic of Korea

**Keywords:** treatment adherence and compliance, patient compliance, medication adherence, digital therapeutics, engagement index, mobile phone

## Abstract

**Background:**

Digital health care apps, including digital therapeutics, have the potential to increase accessibility and improve patient engagement by overcoming the limitations of traditional facility-based medical treatments. However, there are no established tools capable of quantitatively measuring long-term engagement at present.

**Objective:**

This study aimed to evaluate an existing engagement index (EI) in a commercial health management app for long-term use and compare it with a newly developed EI.

**Methods:**

Participants were recruited from cancer survivors enrolled in a randomized controlled trial that evaluated the impact of mobile health apps on recovery. Of these patients, 240 were included in the study and randomly assigned to the Noom app (Noom Inc). The newly developed EI was compared with the existing EI, and a long-term use analysis was conducted. Furthermore, the new EI was evaluated based on adapted measurements from the Web Matrix Visitor Index, focusing on click depth, recency, and loyalty indices.

**Results:**

The newly developed EI model outperformed the existing EI model in terms of predicting EI of a 6- to 9-month period based on the EI of a 3- to 6-month period. The existing model had a mean squared error of 0.096, a root mean squared error of 0.310, and an *R*^2^ of 0.053. Meanwhile, the newly developed EI models showed improved performance, with the best one achieving a mean squared error of 0.025, root mean squared error of 0.157, and *R*^2^ of 0.610. The existing EI exhibited significant associations: the click depth index (hazard ratio [HR] 0.49, 95% CI 0.29-0.84; *P*<.001) and loyalty index (HR 0.17, 95% CI 0.09-0.31; *P*<.001) were significantly associated with improved survival, whereas the recency index exhibited no significant association (HR 1.30, 95% CI 1.70-2.42; *P*=.41). Among the new EI models, the EI with a menu combination of menus available in the app’s free version yielded the most promising result. Furthermore, it exhibited significant associations with the loyalty index (HR 0.32, 95% CI 0.16-0.62; *P*<.001) and the recency index (HR 0.47, 95% CI 0.30-0.75; *P*<.001).

**Conclusions:**

The newly developed EI model outperformed the existing model in terms of the prediction of long-term user engagement and compliance in a mobile health app context. We emphasized the importance of log data and suggested avenues for future research to address the subjectivity of the EI and incorporate a broader range of indices for comprehensive evaluation.

## Introduction

Digital therapeutics (DTx) has the potential to expand accessibility and enhance engagement for patients by addressing the limitations associated with conventional facility-based medical treatments [1]. These interventions have gained considerable attention owing to their effectiveness in addressing various health challenges, which has led to their increasing adoption rate in health care settings [2]. Although DTx offers unique monitoring capabilities, enabling health care providers to remotely track patient progress and tailor interventions, their use remains controversial because of the ambiguity in terms of the DTx’s standpoint and effectiveness [3]. A diverse range of DTx, from smartphone apps for mental health support to wearable devices for chronic disease management, are available to meet the evolving needs of patients and health care providers alike with the availability of real-time and continuous log data for further improvements [4,5]. One such technology that has attracted research interest is mobile health (mHealth), which is used to monitor patients.

In recent years, the use of mHealth technologies in cancer care has steadily increased, offering a promising avenue for improving patient outcomes and revolutionizing health care delivery [6]. With the convenience of the high distribution rate of smartphones of over 86.11% globally, mHealth apps have been increasingly integrated into cancer management, providing patients with remote access to personalized care through physical fitness support, weight management, therapy, information provision, and social support [7,8]. Despite the growing adoption of mHealth solutions in cancer care, existing literature reviews have highlighted a significant challenge, that is, the absence of standardized measures for assessing the use of and compliance with these technologies [9].

The vast majority of studies evaluating intervention engagement rely on either postintervention surveys or interviews [10-13]. Furthermore, when assessing the effectiveness of therapeutic education systems, the methodology often involves twice-weekly clinical checkups and self-reports, despite the pioneering nature of the randomized controlled trial (RCT) for internet-delivered interventions [14]. This highlights the need for a more systematic methodology for evaluating mHealth intervention engagement rather than solely relying on subjective interviews.

This study evaluates the engagement index (EI) in commercial health management apps for long-term use by comparing the newly developed EI model with the existing model.

## Methods

### Study Design

This study aimed to confirm whether a newly developed EI better predicts long-term compliance than an existing EI by using the Web Matrix Visitor Index with modifications, focusing on indices such as click depth, recency, and loyalty based on the Noom app (Noom Inc) usage data. A new menu abundancy index (MI) was introduced, considering the survival time of each menu. In addition, the loyalty index (LI) was enriched by incorporating the final usage week, and the recency index (RI) was refined using permutation entropy to measure the regularity of app usage. This study analyzed data from 233 patients who used the Noom app, part of an RCT involving 960 cancer survivors (breast, colorectal, or lung cancer) aimed at assessing the impact of mHealth apps on recovery. The Noom app, a commercially available weight management tool, was used for its features such as meal logging, step count tracking, weight logging, exercise logging, engagement with health-related content, and messaging.

### Study Population

Data obtained from patients who were recruited from an RCT that investigated the impact of mHealth apps on cancer survivors were used; research aimed to facilitate a smoother recovery for patients with breast, colorectal, or lung cancer as they transition back to their daily lives [15-17]. Written informed consent was obtained from all the participants before study participation. Subsequently, the participants were randomly assigned to 1 of 3 mHealth care groups, including the Noom app group. Of the 960 participants, 233 who used Noom were analyzed for this study.

### Data Collection

Clinical and pathological information related to demographics were extracted from the electronic medical records of patients at the time of recruitment. The data collection was extended up to 18 months after the final patient enrollment, with follow-up assessments scheduled at 3 months and every 6 months after the initial baseline data collection.

Noom, a commercially available mobile app for weight management, can be downloaded from the Google Play Store and the Apple App Store [18]. Distinguished by its distinctive curriculum and human coaching intervention, Noom is a prominent feature in the realm of health and fitness apps [18]. It strives to be a versatile platform for behavioral change, serving as a potent tool for addressing diverse chronic and nonchronic conditions, with the goal of promoting healthier lifestyles for a wider population [19]. Noom has been shown to be an effective mHealth lifestyle platform, with positive results yielded in various clinical scenarios [20-22]. In this study, various features of Noom were used, including, but not limited to, meal logging, step count tracking, weight logging, exercise logging, engagement in health-related content, and messaging functionalities.

### Data Analysis

This study aimed to confirm whether the newly developed EI better predicts long-term compliance than the existing EI. To achieve this, the goal was to predict compliance at 6-9 months or predict survival rates based on that at 3-6 months, with the aim of comparing the performance of the 2 indices. All data analyses were conducted using Python (version 3.8.5; Python Software Foundation).

#### New Engagement Index

At present, there is no established tool to measure engagement in health care apps, thus, we adopted the Web Matrix Visitor Index [23] to effectively measure engagement in the commercial health management app for cancer patients using 7 indices, that are click depth, duration, recency, loyalty, brand, feedback, and interaction. Of the 7 indices, we used 3 (click depth, recency, and loyalty) that were applicable; these could be calculated using the app access log data. Click depth measures the impact of page and event views, whereas recency indicates the speed at which visitors return to the website over time. Specifically, click depth is computed by dividing sessions with a reasonable threshold (eg, 4 pages viewed) by all sessions. Loyalty gauges the extent of long-term interaction with the brand, site, or products. Recency is calculated as 1 divided by the number of days since the most recent session, whereas loyalty is derived by subtracting 1 from the number of visitor sessions during the timeframe from 1.

As the app was not able to provide information when accessed each time, we defined the sessions as 1 day with each index ranging between 0 and 1. Click depth was calculated as the number of weeks with at least 2 menus accessed divided by the number of the current week. Loyalty was calculated as the number of accessed weeks divided by the number of this week. Recency was calculated as 1 over the average number of weeks between visits for each period. Finally, EI was calculated as the average (mean) between the click depth, loyalty, and recency indices.

#### Limitations of Engagement Index

Despite its generalizability, the EI encounters several limitations. First, it may not fully capture all dimensions of user engagement, thus leading to an incomplete representation of user patterns. Second, it is heavily influenced by the natural characteristics of app usage; particularly, over the long term, it can complicate the assessment of long-term app effectiveness. Third, it may fail to account for changes in engagement patterns over time, which limits its applicability in monitoring-maintained user involvement. Finally, its subjective nature could emerge in the metrics when calculating it, thus potentially introducing biases.

#### Calculation of the New Engagement Index

**Figure 1:**
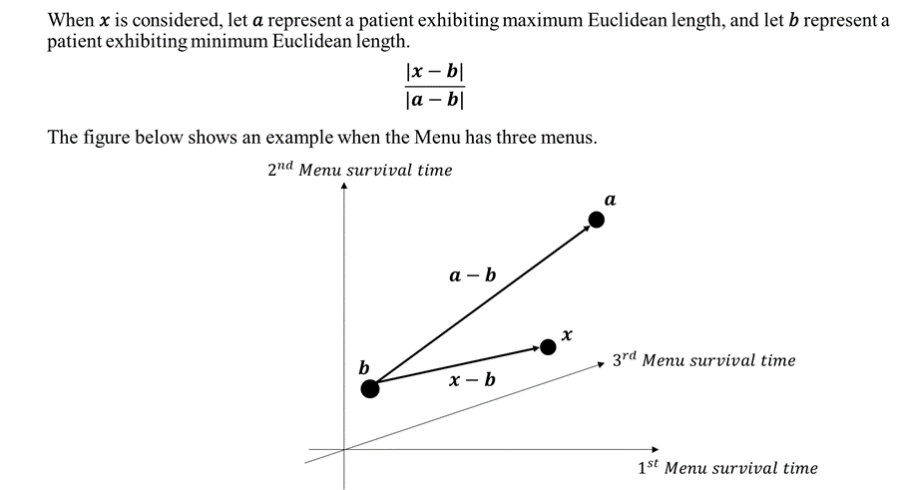
Procedure for calculating the menu abundancy index.

We aimed to enhance EI and its components based on its original characteristics. As the click depth index failed to account for the number of menus available in the app, we introduced the MI, which was devised from the click depth index. The MI considers the different menus offered by the app. By computing the survival time of each menu, with discontinuation defined as continuous nonusage for 45 days, we constructed a vector for each patient, where each co-ordinate represents the survival time of a menu. We chose 45 days as it represents the 75th percentile value of the nonusage period between usages. Subsequently, we computed the Euclidean distance from the origin for each vector. Consequently, menu abundancy is determined as the Euclidean distance between the patient and the patient with the minimum Euclidean distance, divided by the Euclidean distance between the largest and smallest vectors. The diagram and equation used for these calculations are presented in Figure 1.

To enrich the existing LI, which solely considers the number of accessed weeks, we deemed it crucial to incorporate the number of the final weeks used. However, we aimed to prevent this addition from disproportionately influencing the overall index. Therefore, we formulated the LI as the sum of the number of accessed weeks and the natural logarithm of the final usage week number, divided by the total number of weeks used in the study. Due to some resulting values exceeding 1, we applied a simple linear transformation to all values. This involved dividing each value by the maximum value of the LI observed in the study, thus ensuring that the new LI ranged from 0 to 1.

The RI calculates the regularity of app usage. To quantify this, we used permutation entropy (PE), which is a robust time series tool. PE quantifies the complexity of a dynamic system by capturing order relations within a time series and deriving a probability distribution of ordinal patterns [24]. To ensure that PE falls within the range of 0-1, we applied a truncated normal distribution to the values. As lower PE values indicate higher regularity and vice versa, we subtracted the value from 1 to align with the existing RI, where a higher value corresponds to more regular visits. This adjustment maintains consistency with the mathematical representation of the RI.

#### Assumptions

We measured the effectiveness of the newly developed EI through multiple linear regression and survival analysis. These statistical methods were selected to provide deep insights into the interpretability and statistical significance of predictors.

Multiple linear regression reveals the linear relationships between the dependent variable (ie, EI) and the multiple independent variables. Considering these multiple independent variables as predictors could provide a better understanding of the multidimensional nature of usage engagement influenced by factors. Furthermore, this approach facilitates the identification of the most important drivers of long-term engagement, thereby contributing to the development of a more reliable and tailored EI capable of capturing the nuances of patient behavior and adherence patterns. The multiple linear regression assumptions were evaluated for our model. Linearity between the variables was assessed, with values indicating strong correlations: menu abundancy (0.93), LI (0.93), and RI (0.69), all close to 1. The normality of the residuals was supported by Shapiro-Wilk test results: menu abundancy (0.84), LI (0.83), RI (0.85), and new EI (0.90). However, the independence of residuals, as measured by the Durbin-Watson statistic, showed values of 1.02 (menu abundancy), 1.10 (LI), 0.95 (RI), and 0.88 (new EI2), indicating potential autocorrelation. Given that these indices are derived from the same app usage data, achieving complete independence is inherently challenging.

Multiple linear regression can be expressed in a generalized form, that is, formula 1


(*EI_m:m + 3_* = *β*_0_ + *β*_1_ ⋅ *MI_m:m + 3_* + *β*_2_ ⋅ *LI_m:m + 3_* + *β*_3_ ⋅ *RI_m:m + 3_* + *ε*) **(1)**

where the dependent variable is defined as formula 2


(*EI_m:m + 3_* := (*MI_m:m + 3_ + LI_m:m + 3_ + RI_m:m + 3_*)/3) **(2)**

Moreover, *β*_0_ denotes the intercept of the model; *β*_1_ to *β*_3_, the coefficients for the predictors of the MI, LI, and RI, respectively, and *ε*, the error term, which accounts for the variance in the prediction that cannot be explained by the predictors. For regression models, we set 2-month durations, that is, 3-6 and 6-9 months; hence, the values of *m* for each model are 3 and 6, respectively.

Survival analysis, particularly through Cox regression, shows the discontinuation timing of app usage over a specified period. This method accounts for censored data and provides hazard ratios (HRs), quantifying the effect of different predictors on the likelihood of continued app usage. Thus, this approach helps identify which aspect of patient usage pattern is the most predictive and significant for long-term compliance.

Formula 3,


(*h*(*t*) = *h*_0_ (*t*)*exp*(*β*_1_ ⋅ *MI*_3:6_ + *β*_2_ ⋅ *LI*_3:6_ + *β*_3_ ⋅ *RI*_3:6_)) **(3)**

where *h*(*t*) denotes the hazard function at time *t* for predicting the survival days of each app user and *h*_0_ (*t*) denotes the baseline hazard function, which is the hazard for an individual when all the covariates are 0.

### Ethical Consideration

This study was approved by the Institutional Review Board of Asan Medical Center, Korea (Institutional Review Board 2021-1631). Stringent measures were in place to protect the privacy and confidentiality of study data, including secure storage within the hospital premises.

## Results

### Demographic Traits

The demographic characteristics of the study cohort are presented in Table 1. The average age of the patients was 53.55 years, with women constituting approximately two-thirds of the cohort. Each group of cancer type comprised a comparable number of participants, albeit colorectal cancer cases slightly outnumbered the others. Over half of the patients were diagnosed with stage 1, and approximately two-thirds had not undergone chemotherapy. The average BMI of the patients was 23.96 (SD 3.28) kg/m^2^.

**Table 1:** Demographics of participants (N=233).

Demographics		Values
Age (years), mean (SD)		53.55 (10.35)
**Gender** **, n (%)**		
	Men	80 (34)
	Women	153 (66)
**Cancer type** **, n (%)**		
	Breast	78 (33)
	Colorectal	86 (37)
	Lung	69 (30)
**Cancer stage** **, n (%)**		
	0	19 (8)
	I	131 (56)
	II	47 (20)
	III	36 (15)
**Chemotherapy** **, n (%)**		
	Yes	73 (31)
	No	160 (69)
BMI (kg/m^2^), mean (SD)		23.96 (3.28)
**Living** **, n (%)**		
	With family	211 (90)
	Alone	20 (9)
	Other	2 (1)
**Education** **, n (%)**		
	Less than high school	22 (10)
	High school graduate	82 (35)
	College graduate or above	129 (55)
**Job** **, n (%)**		
	Employed	140 (60)
	Unemployed	93 (40)

### Evaluation Between the Existing Engagement Index and New Engagement Index

#### Predicting 6-9 Months Engagement Index Based on the 3-6 Months Engagement Index

To predict the EI of patients with cancer between 6 and 9 months based on their EI between 3 and 6 months, we excluded the initial 0- to 3-month period as the patients were actively under hospital surveillance with ongoing follow-ups. For the existing EI, we observed a mean squared error (MSE) of 0.096, root mean squared error (RMSE) of 0.310, and *R*^2^ of 0.053. For the new EI, we conducted 3 multiple linear regressions to identify the most significant menu combinations. The first combination (new EI1), comprising meal log, exercise log, message sent to the app, reading content, and weight log, exhibited an MSE of 0.036, RMSE of 0.190, and *R*^2^ of 0.511. The second combination (new EI2), involving meal log, exercise log, weight log, and step count login, showed improved performance with an MSE of 0.025, RMSE of 0.157, and *R*^2^ of 0.610. The third combination (new EI3), encompassing meal log, exercise log, message sent to the app, reading content, weight log, and step count login, yielded an MSE of 0.042, RMSE of 0.205, and *R*^2^ of 0.374. The values of the multiple linear regression are presented in Table 2.

**Table 2:** Multiple linear regression results for the existing engagement index and the new engagement index.

	MSE^a^	RMSE^b^	*R^2^*
Existing EI^c^	0.096	0.310	0.053
New EI1	0.036	0.190	0.511
New EI2	0.025	0.157	0.610
New EI3	0.042	0.205	0.374

^a^MSE: mean squared error.

^b^RMSE: root mean squared error.

^c^EI: engagement index.

#### Predicting Survival Rate From 3 to 6 Months

When predicting app usage survival using the individual index of the EI from 3 to 6 months through Cox regression, the existing EI exhibited a log rank test result of *P*<.05. The results indicated a significant association between click depth and loyalty indices, while the RI showed no significance. The click depth index exhibited an HR of 0.49 with a *P* value <.001, which indicates that a higher click depth index is significantly associated with the reduced hazard, thus yielding better outcomes. Similarly, the LI showed an HR of 0.17 and a *P* value <.001, demonstrating a strong and significant association with reduced hazard. Conversely, the RI showed an HR of 1.30 with a *P* value of .41, indicating no significant association. All the log rank test results were statistically significant. The values of the existing EI are presented in Table 3.

**Table 3:** Results of the survival rate prediction using the existing engagement index.

	HR^a^ (95% CI)	*P* value
Click depth index	0.49 (0.29-0.84)	<.001
Loyalty index	0.17 (0.09-0.31)	<.001
Recency index	1.30 (1.70-2.42)	.41

^a^HR: hazard ratio.

For the new EI, we conducted 3 Cox regressions based on the three devised menu combinations. MI 1 incorporates the menus intended for active app users, encompassing those necessitating self-logging. It specifically encompasses meal log, exercise log, messages sent to the app, reading content, and weight log. MI 2 comprises menus available in the app’s free version. It consists of a meal log, exercise log, weight log, and step count login. MI 3 includes all available menus, such as meal log, exercise log, message sent to the app, reading content, weight log, and step count login. Hence, 3 new EIs were created (new EI1, new EI2, and new EI3), which includes each MI (MI1, MI2, and MI3).

New EI1 exhibited no significant association with the MI (HR 0.92; *P*=.81). However, it showed a strong and significant association with the LI (HR 0.28; *P*<.001). Furthermore, it showed a significant association with the RI (HR 0.48). Meanwhile, new EI2 exhibited a similar trend to new EI1, showing no significant association with the MI (HR 0.79; *P*=.50). However, it showed a strong and significant association with the LI (HR 0.3). Moreover, it exhibited a significant association with the RI (HR 0.47). Finally, new EI3 showed no significant association with the MI (HR 0.95; *P*=.82). However, it showed a significant and strong association with the LI (HR 0.26; *P*<.001), but it did not exhibit a significant association with the RI (HR 0.74; *P*=.23).

The MI did not exhibit a significant association with any of the new indices, whereas the LI showed a strong and significant association with all 3 indices. The RI was significantly associated with new EI1 and new EI2 but not with new EI3. All the log rank test results were significant for all the new indices. The values of the new EI are presented in Table 4.

**Table 4:** Survival analysis result for the 3 new engagement indices.

		New EI1	New EI2	New EI3
**Menu abundancy index**				
	HR^a^ (95% CI)	0.92 (0.48-1.77)	0.79 (0.40-1.56)	0.95 (0.57-1.58)
	*P* value	.81	.50	.82
**Loyalty index**				
	HR (95% CI)	0.28 (0.14-0.54)	0.31 (0.16-0.62)	0.26 (0.15-.46)
	*P* value	<.001	<.001	<.001
**Recency index**				
	HR (95% CI)	0.48 (0.28-0.81)	0.47 (0.30-0.75)	0.74 (0.45-1.22)
	*P* value	<.001	<.001	.23

^a^HR: hazard ratio.

## Discussion

### Principal Findings

We evaluated the existing EI in a commercial health management app for long-term use and compared it with the new EI. We evaluated the new EI by first predicting the EI of the 6- to 9-month period based on the EI of the 3- to 6-month period through multiple linear regression and by predicting the survival rate using the EI of the 3- to 6-month period. In both predictions, the new EI exhibited better performance than the existing EI, although the difference was marginal. Moreover, when the RI, the index that best represents the long-term use, was applied in the new EI, a statistically significant difference increased compared with the RI in the existing EI.

### Comparison With Previous Work

Retention has been inconsistently measured across studies in the aspect of mHealth. For instance, a previous study [25] defined retention as continuous use of the app for 6 months after the first use, specifically between 150 and 210 days. Another study measured retention based solely on the number of logs [26]. In addition, 1 study [27] measured retention through follow-up interviews conducted 6 months post intervention. These variations highlight the lack of a standardized retention strategy in mHealth research, posing a significant limitation as results may hinge on a single participant’s interview response rather than reflecting overall trends and maintained use.

While the use of mHealth has the potential to enhance adherence to chronic disease management, research predominantly focuses on the assessment of the usability, feasibility, and acceptability of such apps rather than the direct measurement of adherence [28]. Similarly, studies addressing patient engagement in mHealth interventions in heart failure cases are often underreported and lacking consistency [29]. Moreover, a pressing need to evaluate user engagement in smartphone apps targeting other significant risk factors for cardiovascular disease, such as dietary behaviors, has been emphasized. Yang et al [30] identified 3 key issues concerning the measurement of adherence in mHealth programs. These include challenges in defining and measuring adherence, a tendency for adherence measurements to be grounded in empirical evidence or established theory, and the recognition that adherence is a multifaceted concept, thus requiring a comprehensive assessment rather than reliance on a 1-dimensional approach [30].

Although existing methodologies for measuring adherence to mHealth are limited, fewer measures of adherence with numerical results. Therefore, measurement using the EI has been considered a methodology that could be generally used and numerically measured. Taki et al [31] conducted a study that used the EI to measure engagement in the mHealth app. They used the click depth, loyalty, interaction, recency, and feedback indices and categorized the results into 3 groups to observe changes in the EI over time. However, they noted that some features were not measured by the EI, which may result in the underestimation of engagement of the participants. Similarly, White et al [32] used the EI to examine the demographic differences among 3 groups formed by the EI and used the reading, loyalty, interaction, recency, and feedback indices. However, they were unable to detect an association between the level of engagement and the duration of exclusive breastfeeding, which was possibly due to the limitations of the EI. Furthermore, Schepens Niemiec et al [33] used the loyalty, interaction, usability, and sentiment feedback indices with semistructured interviews to measure app engagement. They acknowledged that as only 4 indices were used, the statistical norm could not be determined to validate the evaluation of the mHealth apps. Despite its applicability to various programs offered by mHealth apps, EI exhibited similar limitations in each study, thereby raising uncertainties regarding its implications. However, despite the thorough investigation, with its simple characteristics, EI can effectively measure engagement in mHealth apps.

Reliance on postintervention surveys or interviews was common in other previous studies evaluating DTx engagement [10-14]. Alternatively, engagement with DTx was occasionally assessed simplistically, such as by marking the first date of a 28-day period without any data upload or by calculating the percentage of participants who completed follow-up at 8 weeks [34,35]. A review of various literature revealed that a more objective measure was evidently needed to evaluate patient engagement in DTx. Although valuable, manual interviews are difficult to replicate and are time consuming due to their labor-intensive nature, involving multiple coordinators. Therefore, the proposal and evaluation of an EI for DTx could enhance the quality of research in this field.

### Limitations

This study represents the inaugural attempt to evaluate the existing EI. While the effectiveness of the index has not yet been evaluated, we have established its reliability despite the comprehensive evaluation for potential upgrades. Furthermore, we were able to demonstrate the importance of log data from a research viewpoint as well as its objectivity, reproducibility, and potential for use to evaluate adherence to mHealth.

EI has a subjective nature in the metrics that may potentially introduce biases, which cannot be overcome despite the update of the index. Furthermore, although the existing EI comprises 7 indices, this evaluation focused only on 3 indices due to the specific characteristics of the app under scrutiny. Also, while the results may indicate that the newly developed EI outperforms the existing EI, the calculation of the existing EI may be simpler than the newly developed EI. However, we believe that this approach is more effective in predicting and representing long-term use.

### Conclusions

This study evaluated the new EI within the commercial health management app by comparing it with the existing EI. Despite thorough evaluation using 2 approaches (forecasting the EI of the 6- to 9-month period based on the EI of the 3- to 6-month period through multiple linear regression and predicting survival rates based on the EI of the 3- to 6-month period), the new EI exhibited a slightly superior performance to the existing EI in both approaches. Although the existing EI appeared too simplistic for evaluating mHealth app adherence, we were able to demonstrate that it effectively reflected adherence without the need for complex calculations, similar to the new EI.

## Abbreviations

DTx: digital therapeutics
EI: engagement index
HR: hazard ratio
LI: loyalty index
mHealth: mobile health
MI: menu abundancy index
MSE: mean squared error
PE: permutation entropy
RCT: randomized controlled trial
RI: recency index
RMSE: root mean squared error

## Data Availability

The data sets generated or analyzed during this study are not publicly available due to the need for institutional review board approval and privacy considerations but are available from the corresponding author upon reasonable request.
